# Supplementary material for: The bat influenza A virus subtype H18N11 induces nanoscale MHCII clustering upon host cell attachment
Source: Nat Commun. 2025 Apr 25;16:3847. doi: 10.1038/s41467-025-58834-y (PMC12032283; doi:10.1038/s41467-025-58834-y)
Supplement: Supplementary file 1 — Supplementary Information [file 41467_2025_58834_MOESM1_ESM.pdf]

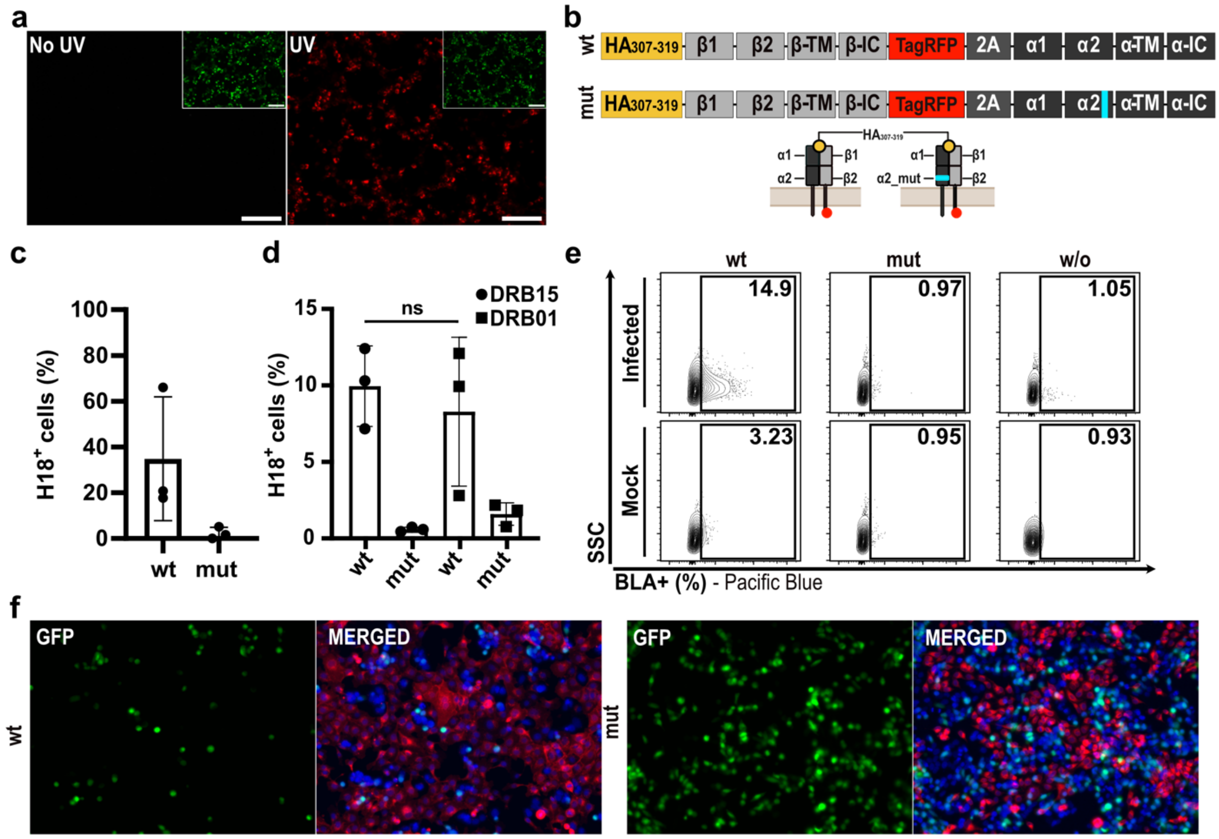

Supplementary Figure 1: Validation of fluorescently-labeled MHCII. (a) HEK293T cells were transiently transfected with $\text{MHCII}_{\text{mEos}}$ and exposed for 20 min under UV light (365 nm) to test for photoconversion of the mEos3.2 fluorophore. Scale bar 100 µm. **(b)** Schematic representation of the fluorescently labeled human MHCII constructs (HLA-DR): $\text{MHCII}_{\text{TagRFP}}$ and $\text{MHCII}_{\text{TagRFPmut}}$. The Influenza HA307-319 peptide was fused to the N-terminus of the beta chain. The red fluorescent TagRFP was fused to the C-terminus of the MHCII beta chain. $\text{MHCII}_{\text{TagRFP}}$ comprises an 11 amino acid substitution in the alpha2 subunit (blue). **(c)** Infection rate of MDCK-II stably expressing $\text{MHCII}_{\text{TagRFP}}$ or $\text{MHCII}_{\text{TagRFPmut}}$ at 24 hours post-infection with H18N11 determined by flow cytometry. *n*=3 independent experiments. Bars indicate mean ± SD **(d)** Infection rate of HEK293T cells transiently transfected with $\text{MHCII}_{\text{TagRFP}}$ and $\text{MHCII}_{\text{TagRFPmut}}$ of the indicated haplotype determined at 24 hours post infection with H18N11 determined by flow cytometry. *n*=3 independent experiments. Bars indicate mean ± SD, Unpaired two-tailed Student's t-test was used for pairwise comparison. ns: not-significant **(e)** Representative flow cytometry plots illustrating β-lactamase activity in cells expressing $\text{MHCII}_{\text{TagRFP}}$, $\text{MHCII}_{\text{TagRFPmut}}$, or no MHCII. Numbers within the plots indicate the percentages of β-lactamase positive cells. *n*= 6. **(f)** Negative control of pH-induced polykaryon formation. HEK293T cells transiently expressing empty vector and GFP were co-cultured with MDCK-II stably expressing $\text{MHCII}_{\text{TagRFP}}$ (wt) or $\text{MHCII}_{\text{TagRFPmut}}$ (mut). Representative images from *n*= 3 independent experiments. Scale bar 100 µm. (c and d) Source data are provided as a Source Data file.

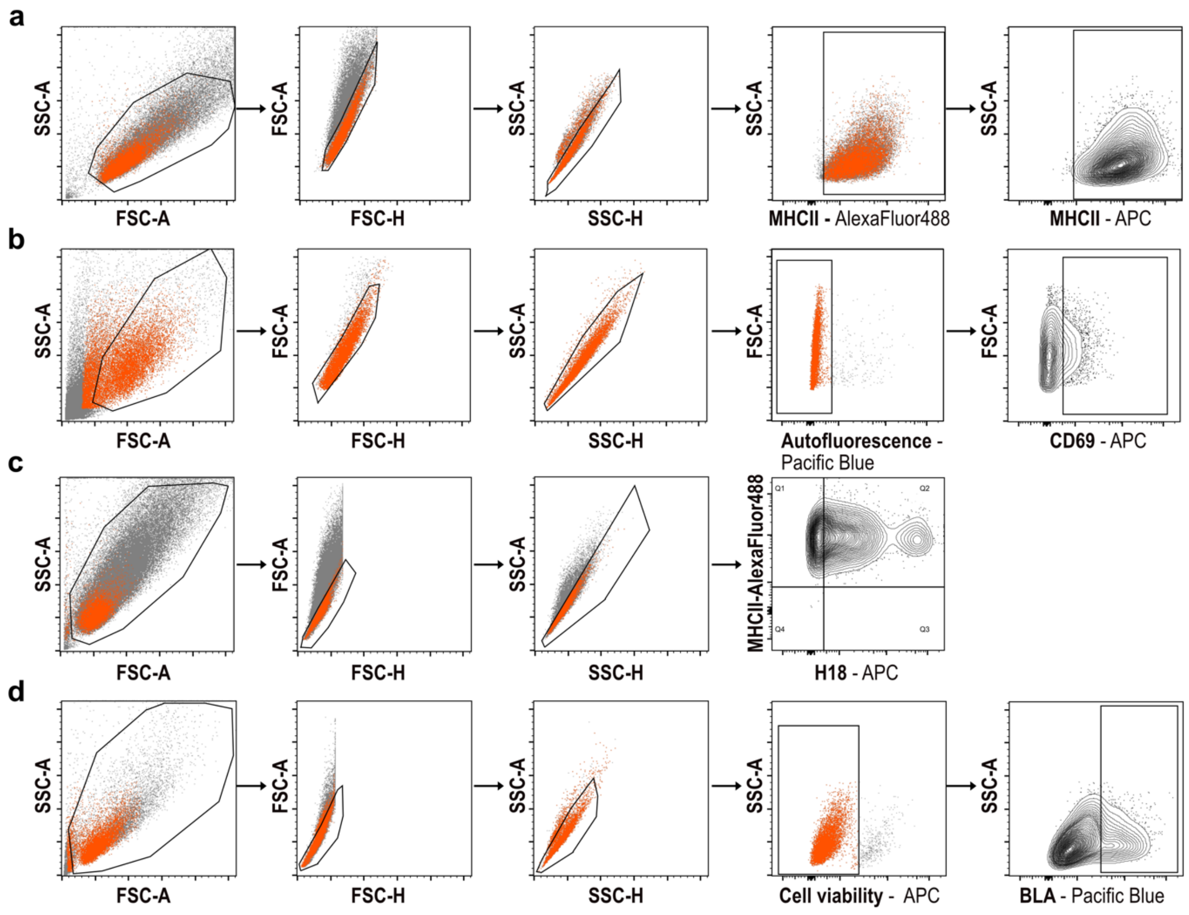

Supplementary Figure 2: Flow cytometric gating strategy for the quantification of MHCII surface expression, T cell activation, virus entry and β-lactamase activity. (a-d) Representative flow cytometry plots showing the gating strategies applied to determine $MHCII_{mEos}$ and $MHCII_{mEosmut}$ surface expression in Fig. 1c (a), T cell activation in Fig. 1d (b), H18N11 infection rates in Fig. 1e (c) and β-lactamase activity in Fig. 1f (d). In (b), FSC-A vs. Pacific Blue gating was performed to exclude autofluorescent cells. Cells staining positive for the amine reactive cell viability dye (Zombie-NIR) were considered dead. FSC, forward scatter; MHCII, major histocompatibility complex class II; APC, Allophycocyanin; SSC, side scatter.

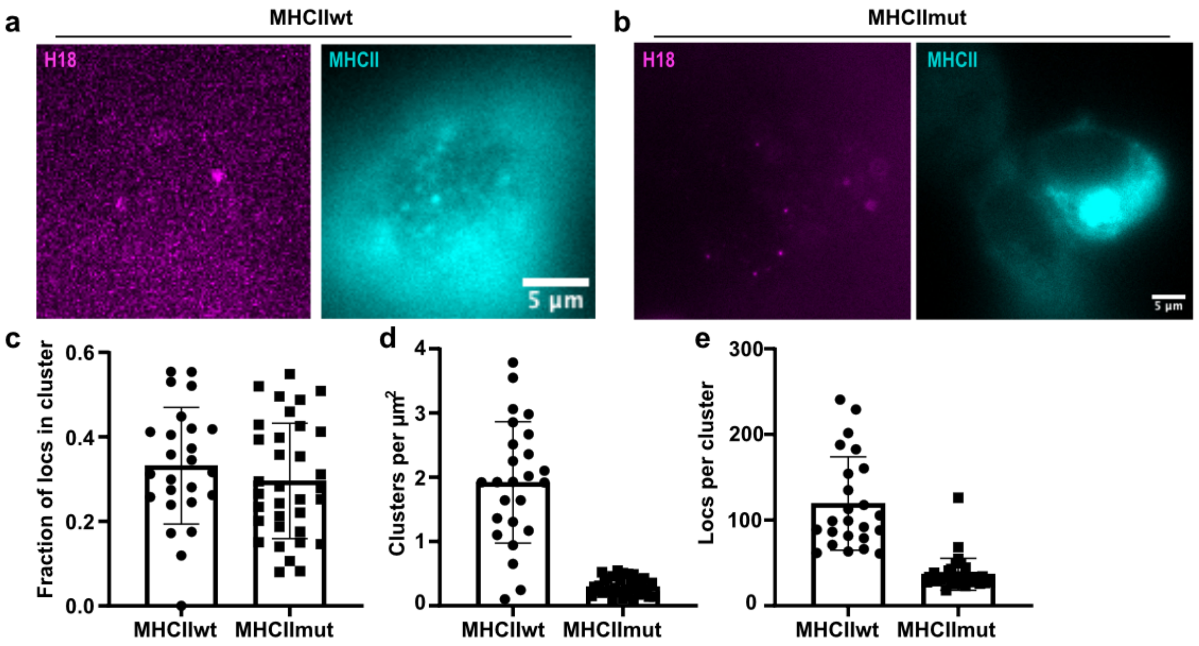

Supplementary Figure 3: Post-fixation analysis of MHCII clusters Representative widefield images of H18N11 viral particles (magenta) and MDCK-II cells stably expressing $MHCII_{mEos}$ **(a)** or $MHCII_{mEosmut}$ **(b)** (cyan), post-fixation, prior to PALM acquisition. Scale bars: 5 µm. Detected localizations were processed and clusters identified using DBSCAN as described in the methods section. Per processed field of view, we calculated the fraction of clustered localizations (Bars indicate mean ± SD; MHCIIwt n=25, MHCIImut n=34) **(c)** and the density of cluster per area (Bars indicate mean ± SD; MHCIIwt n=25, MHCIImut n=34) **(d)**. For each cluster, we also determined the number of localizations, which gives an indication about the local protein density per cluster (Bars indicate mean ± SD; MHCIIwt n=24, MHCIImut n=34) **(e)**.

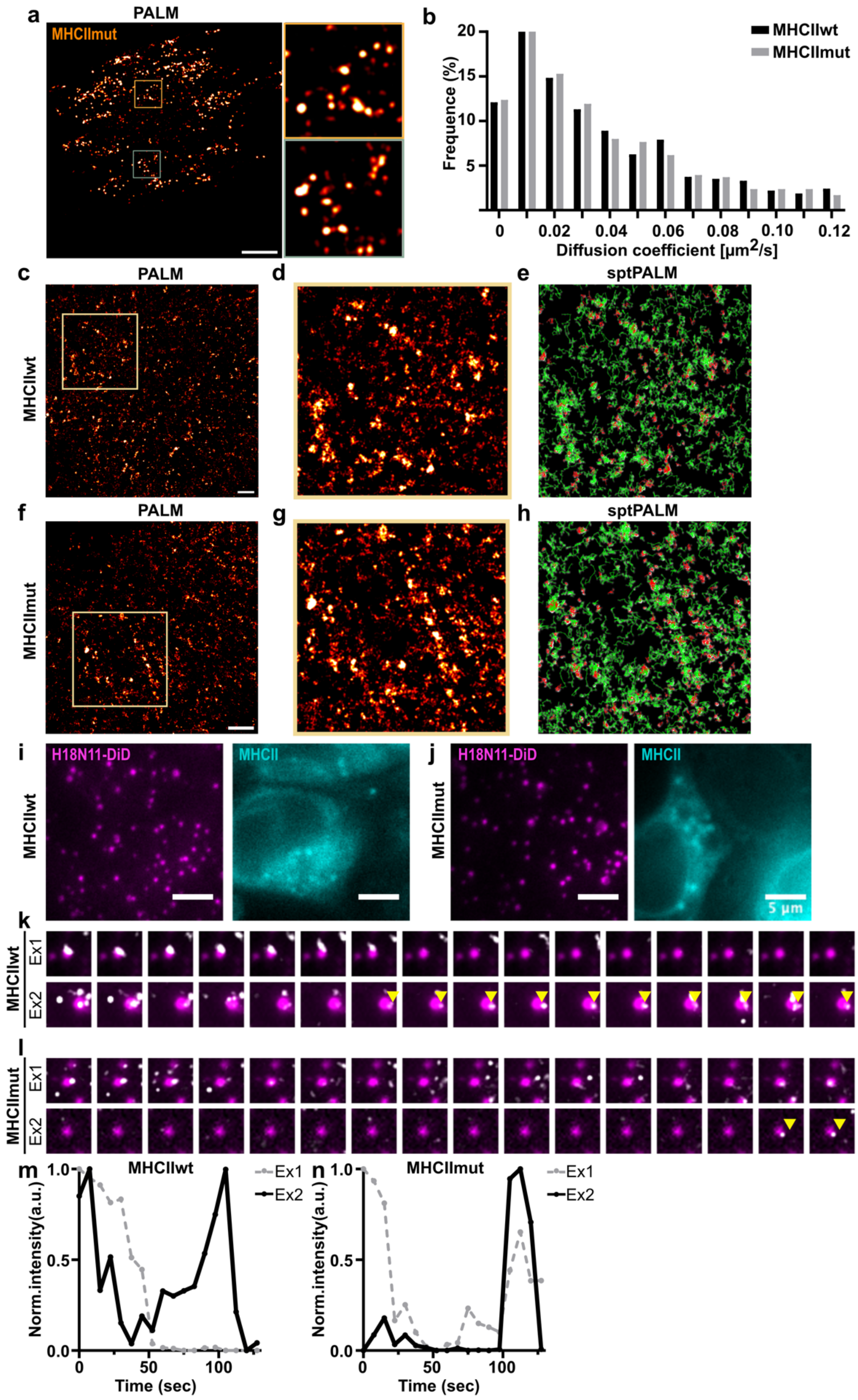

Supplementary Figure 4: MHCII$_{mEos}$ and MHCII$_{mEosmut}$ organize and behave similarly when expressed in MDCK-II cells. To explore if MHCII$_{mEos}$ and MHCII$_{mEosmut}$ are differently organized in uninfected MDCK-II cells, we imaged the apical surface of fixed cells and the basolateral surface of live cells using PALM. Imaging fixed cells **(a)** reveals a similar cluster organization of MHCII$_{mEosmut}$ as shown

for $MHCII_{mEos}$ in Fig. 3B. The cluster size quantification is shown in Fig. 3C. sptPALM of $MHCII_{mEos}$ and $MHCII_{mEosmut}$ revealed similar diffusion coefficients **(b)** and organization of mobile and immobile proteins **(c-h)**. (c and f) show a rendering of a live cell sptPALM acquisition of $MHCII_{mEos}$ (wt) and $MHCII_{mEosmut}$ (mut), respectively, revealing clusters as observed in fixed cells (a), and Fig. 3b. d and g show the highlighted areas in (c) and (f). **(e and h)** To visualize the distribution of mobile and immobile receptors, trajectories with $D<0.01$ µm^2/s were considered as immobile and colored in red. Trajectories of mobile receptors are shown in green. Again, a similar distribution can be observed of $MHCII_{mEos}$ and $MHCII_{mEosmut}$. Scale bars: (a), 1 µm; (c), 1.3 µm; (f), 1.2 µm. (**i and j**) show representative widefield images of $MHCII_{mEos}$ (wt) and $MHCII_{mEosmut}$ (mut) prior to sptPALM acquisition. **(k-n)** Time-binned rendering of MHCII localizations from live-cell acquisitions. MHCII-mEos3.2 (wt and mut) localizations from live-cell acquisitions were rendered using 6 sec time binning (200 frames) resulting in 18 reconstructions across a 5 min acquisition. The position of the labelled IAV particle is shown in magenta, the rendered PALM reconstructions in grey. Recurrent appearance of MHCII clusters (yellow arrow heads) can be observed indicating dynamic exchange of MHCII between the virus-cell interface and the remaining plasma membrane. (k) and (l) show two examples for $MHCII_{mEos}$ (wt) and $MHCII_{mEosmut}$ (mut). (**m and n**) show the corresponding development of the integrated intensity of the rendered PALM localizations over time.

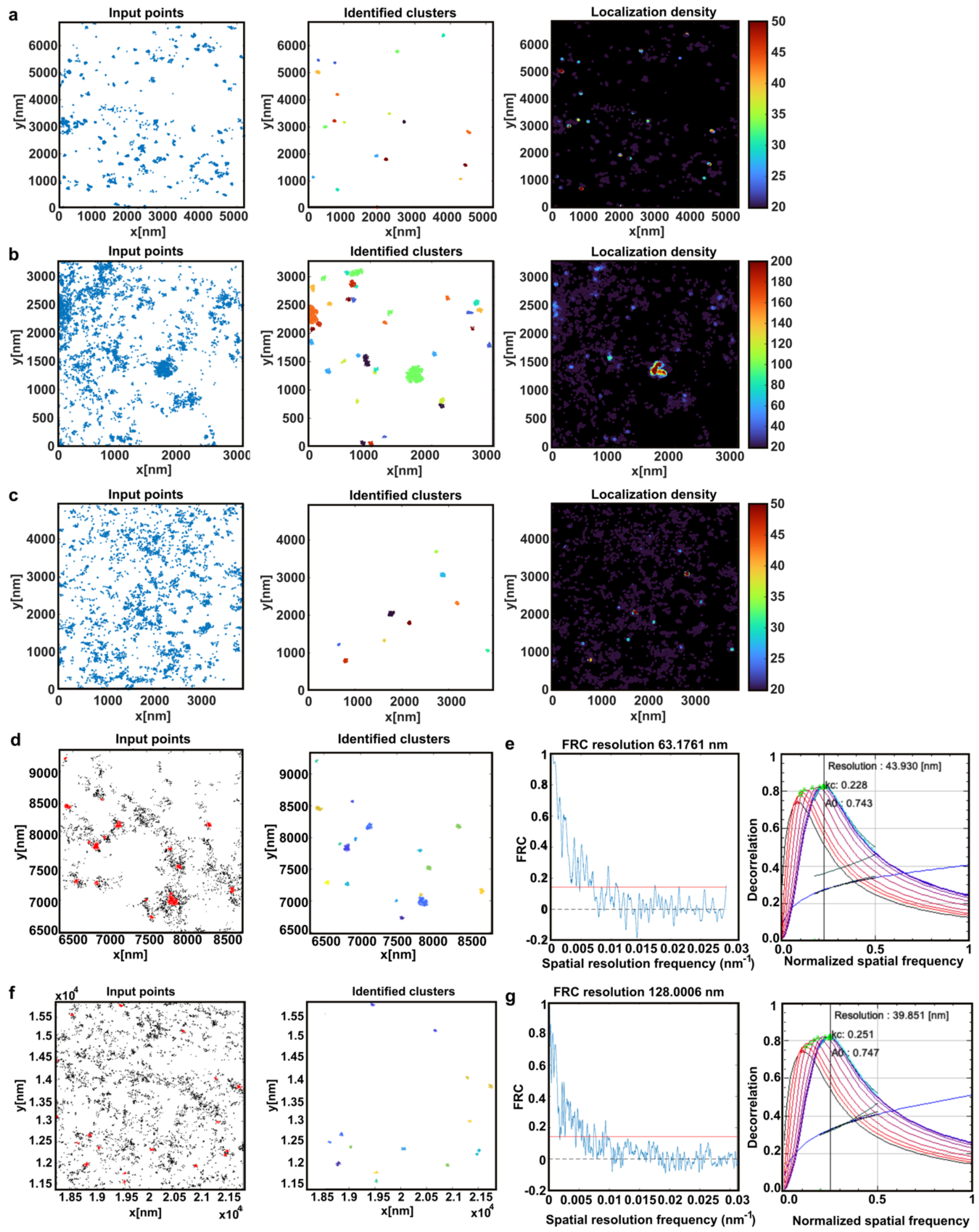

Supplementary Figure 5. Cluster analysis and resolution estimation of single-molecule localization data. MDCK-II cells stably expressing MHCII$_{mEos}$ or MHCII$_{mE\text{-}osmut}$ were imaged using PALM. Representative localization data and extracted clusters for MHCII$_{mEos}$ are shown in **(a-c)** together with a plot showing the local localization density color-coded **(right side).** Shown are representative clustering results for small or large MHCII$_{mEos}$ clusters (a, b) and clusters of MHCII$_{mEosmut}$ (c). The localization density was calculated for each localization as the number of other localizations within a radius of 30 nm (1.5-fold lateral localization precision) to aid the visual identification of clusters. Cluster analysis is described in the methods section. We further analyzed the resolution of our localization datasets using Fourier ring correlation and image decorrelation. **(e)** shows the resolution analysis of the localization data shown in **(d)**. **(f and g)** show the respective representative localization, clustering and resolution estimation for MHCII$_{mEosmut}$.

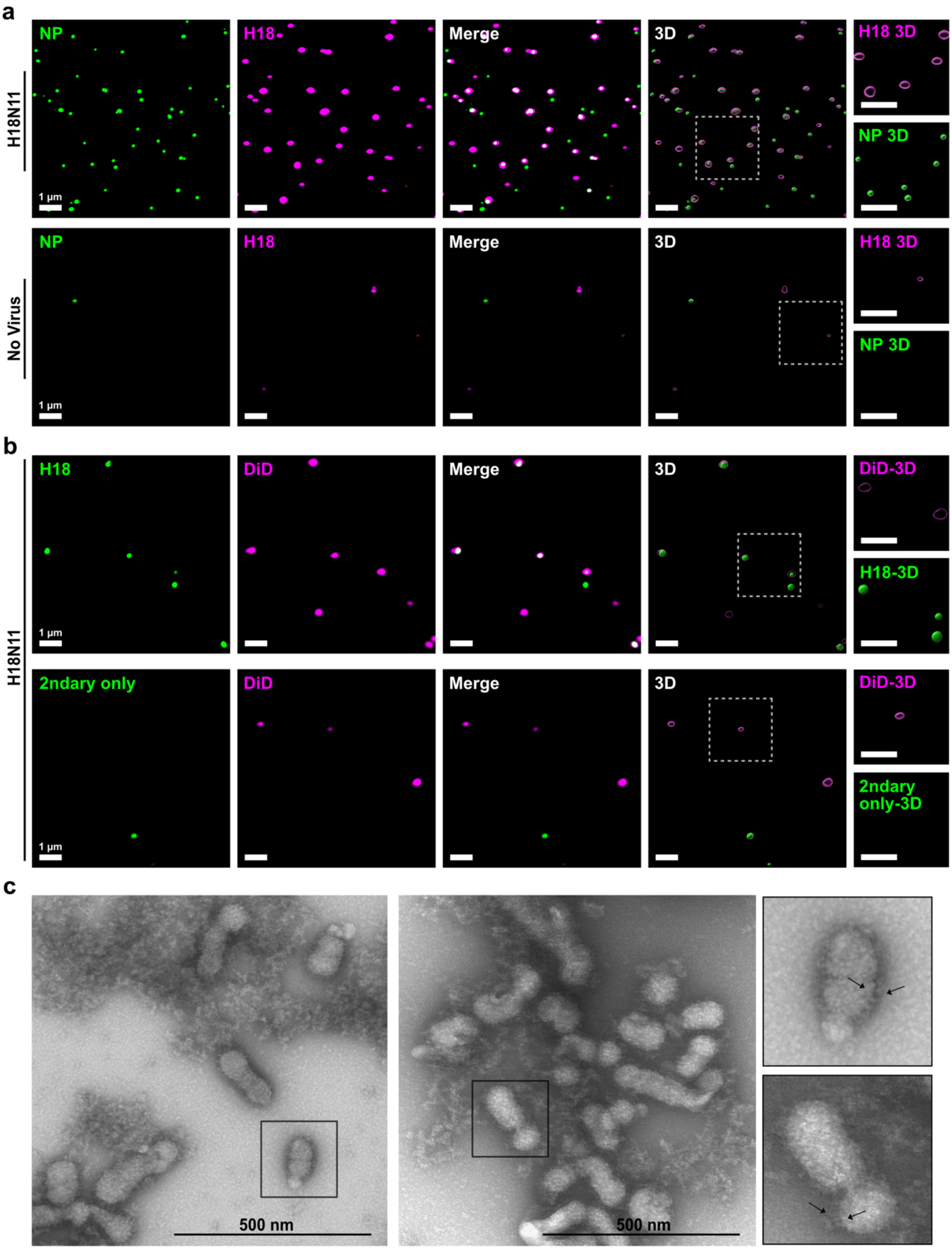

Supplementary Figure 6: Analysis of viral particles within the virus stock preparation. To exclude the presence of exosomes and to ensure that the virus stock preparation is mainly composed of viral particles we performed 3 different stainings. **(a)** Representative images of H18N11 viral particles stained for NP (green) and H18 (magenta). **(b)** Representative images of DiD-labeled H18N11 viral particles (magenta) co-stained for H18 (green). **(c)** Representative images of H18N11 viral particles visualized by transmission electron microscopy (TEM). Black arrows point to H18. Scale bars: (a) and (b), 1 µm; (c), 500 nm. 3D-reconstitution of (a) and (b) are shown in supplementary movies 5 and 6 respectively.

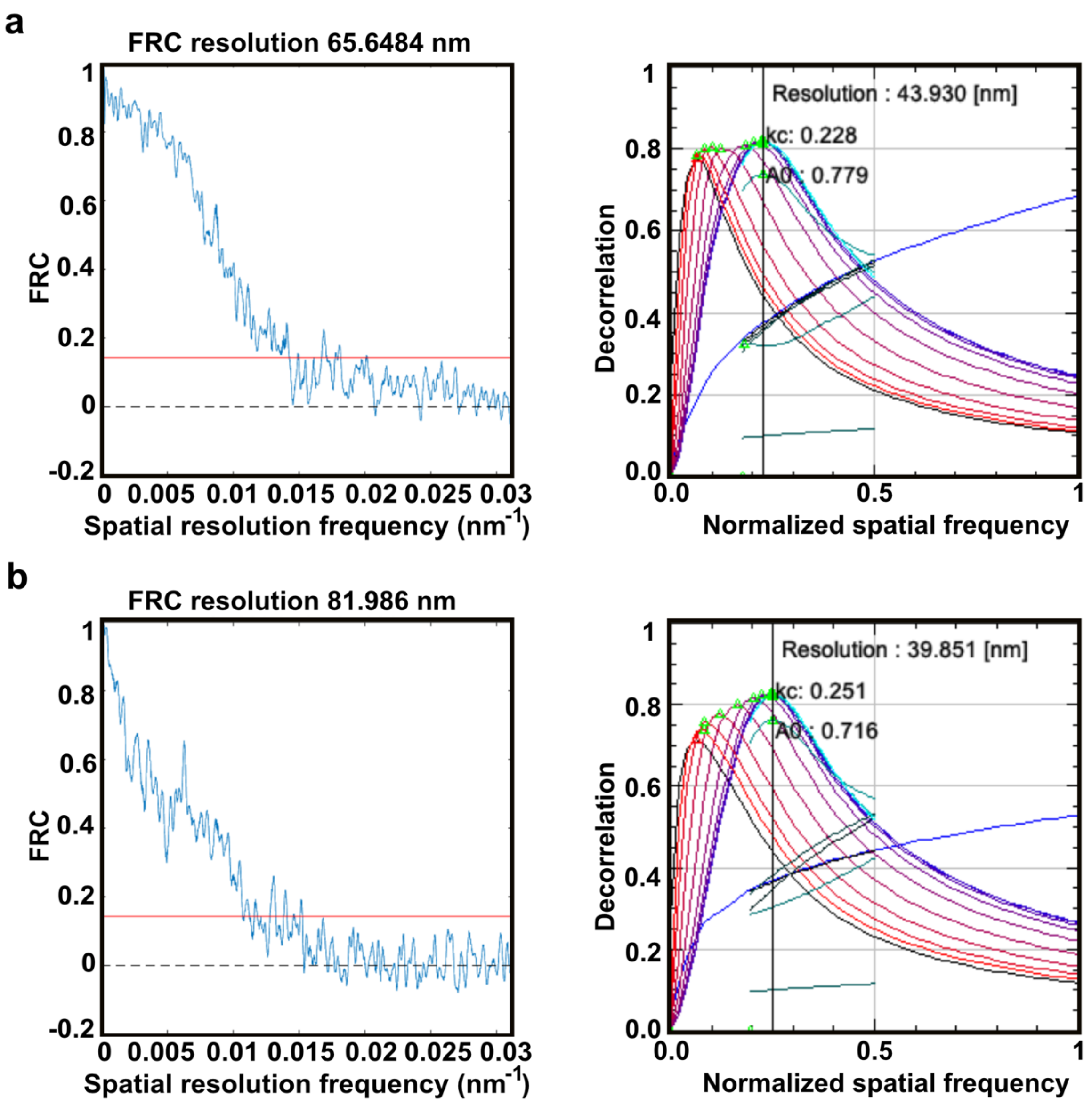

Supplementary Figure 7. Resolution estimation of live-cell sptPALM single-molecule localization data. MDCK-II cells stably expressing MHCII$_{mEos}$ or MHCII$_{mEosmut}$ were imaged using sptPALM. The resolution of our localization datasets was analyzed using Fourier ring correlation and image decorrelation. **(a)** shows the resolution analysis of localization data for MHCII$_{mEos}$, **(b)** for MHCII$_{mEosmut}$.
