# Supplementary material for: The bat influenza A virus subtype H18N11 induces nanoscale MHCII clustering upon host cell attachment
Source: Nat Commun. 2025 Apr 25;16:3847. doi: 10.1038/s41467-025-58834-y (PMC12032283; doi:10.1038/s41467-025-58834-y)
Supplement: Supplementary file 2 — Description of Additional Supplementary Files [file 41467_2025_58834_MOESM2_ESM.docx]

**File Name: Supplementary Movie 1**
Description: Example 1 of MDCK cells stably expressing MHCII_mEos_ imaged using sptPALM. Localizations from a live-cell acquisition were rendered using 6 sec time binning. The position of the labelled IAV particle is shown in magenta. Recurrent appearance of MHCII clusters can be observed indicating dynamic exchanges of MHCII between the virus-interface and the remaining plasma membrane.

**File Name: Supplementary Movie 2**Description: Example 2 of MDCK cells stably expressing MHCII_mEos_ imaged using sptPALM. Localizations from a live-cell acquisition were rendered using 6 sec time binning. The position of the labelled IAV particle is shown in magenta. Recurrent appearance of MHCII clusters can be observed indicating dynamic exchanges of MHCII between the virus-interface and the remaining plasma membrane.

**File Name: Supplementary Movie 3**Description: Example 1 of MDCK cells stably expressing MHCII_mEosmut_ imaged using sptPALM. Localizations from a live-cell acquisition were rendered using 6 sec time binning. The position of the labelled IAV particle is shown in magenta. Recurrent appearance of MHCII clusters can be observed indicating dynamic exchanges of MHCII between the virus-interface and the remaining plasma membrane.

**File Name: Supplementary Movie 4**
Description: Example 2 of MDCK cells stably expressing MHCII_mEosmut_ imaged using sptPALM. Localizations from a live-cell acquisition were rendered using 6 sec time binning. The position of the labelled IAV particle is shown in magenta. Recurrent appearance of MHCII clusters can be observed indicating dynamic exchanges of MHCII between the virus-interface and the remaining plasma membrane.

**File Name: Supplementary Movie 5**
Description: 3D-reconstitution of fluorescently labeled H18N11 viral particles. Viral particles were co-stained for H18 (magenta) and NP (green).

**File Name: Supplementary Movie 6**
Description: 3D-reconstitution of fluorescently labeled H18N11 viral particles. Viral membranes were labeled with DiD (magenta) and co-stained for H18 (green).
