# Supplementary material for: The bat influenza A virus subtype H18N11 induces nanoscale MHCII clustering upon host cell attachment
Source: Nat Commun. 2025 Apr 25;16:3847. doi: 10.1038/s41467-025-58834-y (PMC12032283; doi:10.1038/s41467-025-58834-y)
Supplement: Supplementary file 9 — Reporting Summary [file 41467_2025_58834_MOESM9_ESM.pdf]

nature portfolio

Corresponding author(s): Peter Reuther, Christian Sieben

Last updated by author(s): Mar 11, 2025

Reporting Summary

Nature Portfolio wishes to improve the reproducibility of the work that we publish. This form provides structure for consistency and transparency in reporting. For further information on Nature Portfolio policies, see our Editorial Policies and the Editorial Policy Checklist.

Statistics

For all statistical analyses, confirm that the following items are present in the figure legend, table legend, main text, or Methods section.

n/a	Confirmed	
☐	☒	The exact sample size (*n*) for each experimental group/condition, given as a discrete number and unit of measurement
☐	☒	A statement on whether measurements were taken from distinct samples or whether the same sample was measured repeatedly
☐	☒	The statistical test(s) used AND whether they are one- or two-sided<br>*Only common tests should be described solely by name; describe more complex techniques in the Methods section.*
☒	☐	A description of all covariates tested
☐	☒	A description of any assumptions or corrections, such as tests of normality and adjustment for multiple comparisons
☐	☒	A full description of the statistical parameters including central tendency (e.g. means) or other basic estimates (e.g. regression coefficient) AND variation (e.g. standard deviation) or associated estimates of uncertainty (e.g. confidence intervals)
☐	☒	For null hypothesis testing, the test statistic (e.g. *F*, *t*, *r*) with confidence intervals, effect sizes, degrees of freedom and *P* value noted<br>*Give P values as exact values whenever suitable.*
☒	☐	For Bayesian analysis, information on the choice of priors and Markov chain Monte Carlo settings
☒	☐	For hierarchical and complex designs, identification of the appropriate level for tests and full reporting of outcomes
☒	☐	Estimates of effect sizes (e.g. Cohen's *d*, Pearson's *r*), indicating how they were calculated

Our web collection on statistics for biologists contains articles on many of the points above.

Software and code

Policy information about availability of computer code

Data collection	BD FACSDiva Software v.8.0.1, Zeiss AxioVision 6D Software, Nikon NIS Elements AR 5.11.01, ZEN 3.0 black edition, ThunderSTORM v1.3, previously used custom processing codes (https://github.com/christian-7/)
Data analysis	FlowJo v10.8.1. software, Prism GraphPad 9, Python, Fiji with ImageJ 1.53q, Decode v0.1,Matlab 2020b, TrackPy, Imaris 10.2.0

For manuscripts utilizing custom algorithms or software that are central to the research but not yet described in published literature, software must be made available to editors and reviewers. We strongly encourage code deposition in a community repository (e.g. GitHub). See the Nature Portfolio guidelines for submitting code & software for further information.

Data

Policy information about availability of data

All manuscripts must include a data availability statement. This statement should provide the following information, where applicable:

- Accession codes, unique identifiers, or web links for publicly available datasets
- A description of any restrictions on data availability
- For clinical datasets or third party data, please ensure that the statement adheres to our policy

Source data are provided with this paper. Example datasets are available via https://zenodo.org/records/14251817.

Research involving human participants, their data, or biological material

Policy information about studies with human participants or human data. See also policy information about sex, gender (identity/presentation), and sexual orientation and race, ethnicity and racism.

Reporting on sex and gender	*Use the terms sex (biological attribute) and gender (shaped by social and cultural circumstances) carefully in order to avoid confusing both terms. Indicate if findings apply to only one sex or gender; describe whether sex and gender were considered in study design; whether sex and/or gender was determined based on self-reporting or assigned and methods used. Provide in the source data disaggregated sex and gender data, where this information has been collected, and if consent has been obtained for sharing of individual-level data; provide overall numbers in this Reporting Summary. Please state if this information has not been collected. Report sex- and gender-based analyses where performed, justify reasons for lack of sex- and gender-based analysis.*
Reporting on race, ethnicity, or other socially relevant groupings	*Please specify the socially constructed or socially relevant categorization variable(s) used in your manuscript and explain why they were used. Please note that such variables should not be used as proxies for other socially constructed/relevant variables (for example, race or ethnicity should not be used as a proxy for socioeconomic status). Provide clear definitions of the relevant terms used, how they were provided (by the participants/respondents, the researchers, or third parties), and the method(s) used to classify people into the different categories (e.g. self-report, census or administrative data, social media data, etc.) Please provide details about how you controlled for confounding variables in your analyses.*
Population characteristics	*Describe the covariate-relevant population characteristics of the human research participants (e.g. age, genotypic information, past and current diagnosis and treatment categories). If you filled out the behavioural & social sciences study design questions and have nothing to add here, write "See above."*
Recruitment	*Describe how participants were recruited. Outline any potential self-selection bias or other biases that may be present and how these are likely to impact results.*
Ethics oversight	*Identify the organization(s) that approved the study protocol.*

Note that full information on the approval of the study protocol must also be provided in the manuscript.

Field-specific reporting

Please select the one below that is the best fit for your research. If you are not sure, read the appropriate sections before making your selection.

[x] Life sciences [ ] Behavioural & social sciences [ ] Ecological, evolutionary & environmental sciences

For a reference copy of the document with all sections, see nature.com/documents/nr-reporting-summary-flat.pdf

Life sciences study design

All studies must disclose on these points even when the disclosure is negative.

Sample size	No sample-size calculations were performed. Sample size was determined to be adequate based on the magnitude and consistency of measurable differences between groups.
Data exclusions	No data were excluded
Replication	For all major experiments at least three independent experiments were done and in all cases results could be reproduced. The number of repeats for each experiment is reported in the figure legends.
Randomization	No formal randomization method was used
Blinding	Investigators were not blinded to the experimental analyses, as this was deemed unnecessary for sample processing, the data collection and analysis.

Reporting for specific materials, systems and methods

We require information from authors about some types of materials, experimental systems and methods used in many studies. Here, indicate whether each material, system or method listed is relevant to your study. If you are not sure if a list item applies to your research, read the appropriate section before selecting a response.

Materials & experimental systems

n/a	Involved in the study	
☐	☒	Antibodies
☐	☒	Eukaryotic cell lines
☒	☐	Palaeontology and archaeology
☒	☐	Animals and other organisms
☒	☐	Clinical data
☒	☐	Dual use research of concern
☒	☐	Plants

Methods

n/a	Involved in the study	
☒	☐	ChIP-seq
☐	☒	Flow cytometry
☒	☐	MRI-based neuroimaging

Antibodies

Antibodies used

Monoclonal mouse anti-H18; In house (1:100)
Polyclonal rabbit anti-H18; In house (1:750)
AlexaFluor647 goat anti-rabbit, ThermoFisher, A21244 (1:250) (1:1000)
AlexaFluor488 goat anti-mouse, Dianova, 115546062, (1:1000)
AlexaFluor488 chicken anti-rabbit, Invitrogen, A21441 (1:1500)
APC-labeled anti-mouse, BD Biosciences, 550826 (1:200)
Monoclonal mouse anti-human HLADR, BioLegend, 327002 (1:500)
FITC-labeled anti-human CD3 antibody, clone UCHT1, BioLegend, 317302 (1:200)
APC-labeled anti-CD69, Life Technologies, MHCD6905 (1:200)
Monoclonal mouse anti-H18N11-NP; In house (1:500)
HRP-conjugated anti-mouse antibody, Dianova, 315-035-045 (1:500)

Validation

Monoclonal mouse anti-H18 was validated on H18N11-infected and mock-infected cells
Polyclonal rabbit anti-H18 was validated on H18N11-infected and mock-infected cells
AlexaFluor 647 goat anti-rabbit was validated by omission of the primary antibody
AlexaFluor488 goat anti-mouse was validated by omission of the primary antibody
AlexaFluor488 chicken anti-rabbit was validated by omission of the primary antibody
APC-labeled anti-mouse was validated by omission of the primary antibody
Monoclonal mouse anti-human HLADR was validated with HLA-DR expressing and non-expressing cells
FITC-labeled anti-human CD3 antibody was validated on CD3 non-expressing cells
APC-labeled anti-CD69 antibody was validated on unstimulated T-cells
Monoclonal mouse anti-H18N11-NP was validated on H18N11-infected and mock-infected cells
HRP-conjugated anti-mouse antibody by omission of the primary antibody

Eukaryotic cell lines

Policy information about cell lines and Sex and Gender in Research

Cell line source(s)

HEK293T cells were purchased from the American Type Culture Collection (ATCC); #CRL-3216
MDCKII cells were described previously as clone no.2 (https://doi.org/10.1038/s41586-019-0955-3)
Baby Hamster Kidney Fibroblasts (BHK-21) cells were obtained from the German Cell Culture Collection (DSZM)
CH7C17 Jurkat T cells were kindly provided by Wolfgang Schamel

Authentication

None of the cell lines used were authenticated.

Mycoplasma contamination

Cell lines are regularly screened for mycoplasma contamination. Cells were not contaminated with mycoplasma.

Commonly misidentified lines
(See ICLAC register)

Name any commonly misidentified cell lines used in the study and provide a rationale for their use.

Plants

Seed stocks

Report on the source of all seed stocks or other plant material used. If applicable, state the seed stock centre and catalogue number. If plant specimens were collected from the field, describe the collection location, date and sampling procedures.

Novel plant genotypes

Describe the methods by which all novel plant genotypes were produced. This includes those generated by transgenic approaches, gene editing, chemical/radiation-based mutagenesis and hybridization. For transgenic lines, describe the transformation method, the number of independent lines analyzed and the generation upon which experiments were performed. For gene-edited lines, describe the editor used, the endogenous sequence targeted for editing, the targeting guide RNA sequence (if applicable) and how the editor was applied.

Authentication

Describe any authentication procedures for each seed stock used or novel genotype generated. Describe any experiments used to assess the effect of a mutation and, where applicable, how potential secondary effects (e.g. second site T-DNA insertions, mosiacism, off-target gene editing) were examined.

Flow Cytometry

Plots

Confirm that:

- [x] The axis labels state the marker and fluorochrome used (e.g. CD4-FITC).
- [x] The axis scales are clearly visible. Include numbers along axes only for bottom left plot of group (a 'group' is an analysis of identical markers).
- [x] All plots are contour plots with outliers or pseudocolor plots.
- [x] A numerical value for number of cells or percentage (with statistics) is provided.

Methodology

Sample preparation	see Online Methods for details<br><br>MHCII surface expression:<br>MDCK-II cells stably expressing MHCIImEos or MHCIImEosmut were trypsinized, centrifuged and stained for anti-HLADR.<br><br>Infection of MHCII expressing MDCKII cells with H18N11:<br>MHCIImEos or MHCIImEosmut cells were infected at an MOI of 5 for 1 h at 37°C. The inoculum was replaced with growth medium and cells were incubated for 24 hours at 37°C. After incubation, cells were washed, trypsinized and fixed with 4% PFA. Cells were then washed and stained for anti-H18.<br><br>T cell activation:<br>BHK-21 were seeded and transfected with MHCIIwt or MHCIImut. 24 hpt the transfected cell were co-cultured with CH7C17 Jurkat T cells. 6 hours post co-culture cells were stained and analyzed with a BD FACSCanto II flow cytometer<br><br>M1-b-lactamase entry assay :<br>HEK293T cells were transfected with pCAGGS-BlaM1, pCAGGS-H18 and pCAGGS-N11. 72 hpt the BlaM1 VLP-containing supernatants were harvested and centrifuged. For detection of M1-b-lactamase activity, MHCII expressng cells were incubated with for 4 hours at 37°C. Cells were loaded with the CCF2-AM substrate for 30 min at 37°C.
Instrument	BD LSRFortessa™ Cell Analyzer , BD FACSCanto II (BD Biosciences)
Software	BD FACSDiva Software v.8.0.1, FlowJo v10.8.1. software
Cell population abundance	Abundance of MHCII surface expressing cells, infected cells, CD69 and b-lactamase positive cells are shown in the relevant figure (Fig. 1)
Gating strategy	FSC-SSC gating was used to determine cell population and eliminate debris. FSC-H and FSC-A, SSC-H and -A gating were used to exclude doublets. Cell types were identified by staining for the respective marker (HLADR, H18, CD69). For b-lactamase entry assay, dead cells were excluded.

- [x] Tick this box to confirm that a figure exemplifying the gating strategy is provided in the Supplementary Information.
